# Management of Peripartum Cardiomyopathy With and Without Bromocriptine: A Comparative Study

**DOI:** 10.7759/cureus.113260

**Published:** 2026-07-23

**Authors:** Joseph Raj, Sheeba Sharon Gnanaiah J., Nishanth Isaac E., J. Karunya Kiran Gnanaiah

**Affiliations:** 1 Department of Cardiothoracic Surgery, Sri Venkateshwaraa Medical College Hospital and Research Centre, Ariyur, IND; 2 Department of Obstetrics and Gynaecology, Tirunelveli Medical College, Tirunelveli, IND; 3 Department of Biochemistry, Thoothukudi Government Medical College, Thoothukudi, IND; 4 Department of Anaesthesia, Government Theni Medical College, Theni, IND

**Keywords:** bromocriptine, left ventricular ejection fraction, mortality, peripartum cardiomyopathy, pregnancy-related heart failure, prolactin

## Abstract

Introduction: Peripartum cardiomyopathy is an uncommon but potentially severe cause of pregnancy-associated systolic heart failure. Bromocriptine has been proposed as an adjunctive therapy because it suppresses prolactin release, but its interpretation depends on the background heart failure regimen, dose, anticoagulation practice, and patient selection. This study compared short-term left ventricular ejection fraction (LVEF) recovery in patients managed with standard therapy with or without low-dose bromocriptine.

Methods: This prospective comparative observational study was conducted at a tertiary care center in South India from October 2020 to October 2021. A total of 50 patients with peripartum cardiomyopathy and LVEF below 45% were analyzed according to treatment received. Group A (n=25) received bromocriptine 2.5 mg once daily for one week in addition to standard pregnancy/postpartum-compatible heart failure therapy, and Group B (n=25) received standard therapy alone. Treatment allocation was not randomized, and no allocation concealment was used. Echocardiography was used to assess LVEF at baseline, discharge, and one month. Continuous variables were compared with the independent samples Student's t-test, and categorical variables were compared with the chi-square test or Fisher's exact test.

Results: Baseline LVEF did not differ significantly between Group A and Group B (38.36 ± 2.40% vs 39.00 ± 1.19%; p = 0.238). Important baseline differences were present, including gestational age at presentation (33.76 ± 7.84 vs 37.72 ± 1.57 weeks; p = 0.017), body mass index (22.49 ± 3.10 vs 24.01 ± 1.75 kg/m2; p = 0.037), comorbidity distribution (p = 0.033), electrocardiographic findings (p = 0.033), and chest radiographic findings (p = 0.004). At discharge, mean LVEF was 47.52 ± 2.52% in Group A and 46.56 ± 2.82% in Group B (p = 0.210). At one month, mean LVEF was higher in Group A than in Group B (57.00 ± 1.71% vs 49.84 ± 1.38%; p < 0.001). Gestational age at delivery (p = 0.031) and birth weight (p = 0.010) differed between the groups; neonatal intensive care unit admission did not (p = 0.560). Recorded Apgar score distributions were retained as abstracted, but the uniform control-group values require cautious interpretation.

Conclusion: Both groups showed improvement in left ventricular function. Low-dose one-week bromocriptine was associated with greater one-month LVEF in this cohort, but the non-randomized design, baseline imbalances, limited documentation of background therapy and anticoagulation, and short follow-up preclude causal claims. The findings should be interpreted as hypothesis-generating and require confirmation in larger protocolized studies with adjustment for confounding and longer follow-up.

## Introduction

Peripartum cardiomyopathy (PPCM) is an idiopathic form of pregnancy-associated systolic heart failure that typically presents toward the end of pregnancy or in the months after delivery and is characterized by reduced left ventricular systolic function, commonly defined by left ventricular ejection fraction (LVEF) below 45% in the absence of another identifiable cause [1,2]. Contemporary pregnancy-specific guidance emphasizes early recognition, echocardiographic confirmation, multidisciplinary care, and treatment that is compatible with pregnancy and lactation, while general heart failure guidance for reduced ejection fraction identifies four foundational drug classes when they are clinically appropriate and not contraindicated [2,3]. Because symptoms such as dyspnea, fatigue, edema, and reduced exercise tolerance may resemble late-pregnancy changes, delayed recognition can increase the risk of pulmonary edema, arrhythmia, thromboembolism, cardiogenic shock, and persistent ventricular dysfunction [4,5].

PPCM is a global disorder with heterogeneous presentation, treatment, and recovery. In the European Society of Cardiology (ESC) EURObservational Research Programme (EORP) PPCM registry, clinical presentation and management varied across regions, and myocardial recovery was incomplete in a substantial proportion of patients at follow-up [6]. A subsequent one-year analysis from the same registry showed continued maternal risk, including mortality, thromboembolic events, and incomplete left ventricular recovery in a clinically important subset [7]. These data support the need for optimized, context-appropriate management strategies while recognizing that treatment effects may be difficult to interpret without comparable baseline risk profiles.

The disease is biologically and clinically heterogeneous. Hypertensive disorders of pregnancy frequently coexist with PPCM and may define a distinct phenotype with different recovery patterns [8]. Genetic susceptibility is also increasingly recognized, with evidence of shared predisposition between PPCM and dilated cardiomyopathy, including truncating variants in genes related to myocardial structure and function [9]. These factors may influence baseline severity, hemodynamic load, and the trajectory of recovery independent of any specific intervention.

One mechanistic hypothesis links oxidative stress to cleavage of prolactin into a 16 kDa fragment with anti-angiogenic and cardiotoxic properties [10]. Bromocriptine, a dopamine receptor agonist that inhibits prolactin release, has therefore been investigated as an adjunct to standard heart failure therapy in PPCM [11]. A German multicenter randomized trial compared one-week and eight-week bromocriptine regimens. Left ventricular ejection fraction improved substantially in both groups, with no statistically significant difference in the change in left ventricular ejection fraction between the two regimens [12]. The previously published rationale-and-design article for the same registered trial (NCT00998556) specified prophylactic-dose anticoagulation during the period of bromocriptine treatment in both groups [13]. An editorial proposed the BOARD acronym for a combined peripartum cardiomyopathy management approach comprising bromocriptine, oral heart-failure therapies, anticoagulants, vasorelaxing agents, and diuretics [14]. A subsequent systematic review and meta-analysis found that prolactin inhibition was associated with improved left ventricular ejection fraction and greater odds of left ventricular recovery, but not with a significant reduction in all-cause mortality; the authors therefore emphasized the need for larger multicenter studies with longer follow-up [15]. Proteomic analyses from contemporary registries further support a role for inflammatory, vascular, fibrotic, and coagulation pathways in PPCM biology [16].

The present study was designed to compare early LVEF recovery among patients with PPCM who received low-dose short-course bromocriptine in addition to standard therapy versus those who received standard therapy alone. The primary objective was to compare LVEF at discharge and one month between treatment groups. Secondary objectives were to describe maternal, obstetric, and neonatal outcomes and to identify baseline factors that may affect interpretation of group comparisons. Serum prolactin was not analyzed because prolactin data were not available in the analyzable dataset.

## Materials and methods

This was a prospective comparative observational study conducted in the Department of Obstetrics and Gynecology, Government Rajaji Hospital and Madurai Medical College, Madurai, Tamil Nadu, India, from October 2020 to October 2021. The study was approved by the Institutional Ethics Committee, Madurai Medical College & Govt. Rajaji Hospital. Informed consent was obtained from participants. The study was not designed or reported as a randomized controlled trial, and no allocation concealment, blinding, or Consolidated Standards of Reporting Trials (CONSORT) flow diagram was applicable to the available dataset.

Study population

Patients were eligible if they had new-onset heart failure with left ventricular systolic dysfunction during pregnancy or within five months postpartum, without prior structural heart disease or another identifiable cause of cardiac dysfunction. Patients with LVEF above 45% or an alternative structural or secondary cardiac cause were excluded.

No a priori sample size calculation was documented. Eligible patients admitted during the study period were enrolled consecutively and analyzed according to treatment received. A total of 50 patients diagnosed with PPCM and LVEF below 45% were included. Patients were grouped as follows: Group A received bromocriptine in addition to standard therapy, and Group B received standard therapy without bromocriptine.

The classical PPCM definition emphasizes onset toward the end of pregnancy or within the postpartum period. The operational dataset used in this study included a small number of patients presenting before 25 weeks of gestation. These patients were retained to preserve the original data, but this broader inclusion window is acknowledged as a potential source of heterogeneity and confounding.

Treatment groups and standard therapy

Group A received bromocriptine 2.5 mg orally once daily for one week. This was a low-dose short-course regimen used in the local clinical protocol during the study period. Published PPCM bromocriptine protocols include short-course dosing as well as higher cumulative dosing, such as 2.5 mg twice daily for two weeks followed by 2.5 mg once daily for six weeks in prolonged regimens; therefore, the present intervention should not be interpreted as equivalent to longer or higher cumulative dose protocols [12,13].

Standard therapy was provided by the treating team and consisted of pregnancy/postpartum-compatible heart failure management as clinically appropriate. The available departmental protocol included oxygen and fluid/salt restriction when indicated, loop diuretics for congestion, beta-blocker therapy when hemodynamically tolerated, vasodilator therapy with hydralazine and nitrates when vasodilation was required during pregnancy or early postpartum, postpartum initiation or transition to angiotensin-converting enzyme (ACE) inhibitor or angiotensin receptor blocker therapy when appropriate, and anticoagulation when indicated by left ventricular dysfunction severity, thrombus, arrhythmia, immobility, or other thromboembolic risk. Contemporary full four-pillar Heart Failure with reduced Ejection Fraction (HFrEF) therapy was not documented for all patients and may not have been applicable during pregnancy or lactation [2,3].

Anticoagulation was assessed as part of standard therapy and was not intended to be restricted to Group A. However, the dataset did not capture patient-level drug names, doses, duration, adherence, or whether every patient receiving bromocriptine also received systematic anticoagulation. This limits conclusions about treatment equivalence and bromocriptine safety.

Outcome assessment

The primary outcome was improvement in LVEF from baseline to discharge and at one month. LVEF was assessed by echocardiography at baseline and during follow-up. Echocardiographic measurements followed established chamber quantification recommendations [17]. Weekly inpatient echocardiographic monitoring was recorded in the study protocol, but the available records did not document whether all examinations were performed by the same observer or whether assessors were blinded to the treatment group.

Secondary outcomes included gestational age at delivery, mode of delivery, birth weight, Apgar scores, neonatal intensive care unit admission, and documented maternal morbidity. Baseline variables included age, parity, gestational age at presentation, body mass index, comorbidities, presenting complaints, past history, family history, laboratory values, electrocardiographic findings, chest radiographic findings, and baseline LVEF.

Statistical analysis

IBM SPSS Statistics for Windows, version 26 (Released 2018; IBM Corporation, Armonk, New York, United States) was used for statistical analysis. Continuous variables were summarized as mean ± standard deviation (SD) and compared using the independent samples Student's t-test. Categorical variables were summarized as frequencies and percentages and compared using the chi-square test or Fisher's exact test, as appropriate. A two-tailed p-value less than 0.05 was considered statistically significant.

Because the sample size was small and several baseline variables differed between groups, the analysis was treated as exploratory. No multivariable adjustment, propensity score method, or correction for multiple comparisons was performed. For the principal LVEF comparisons, unadjusted mean differences with 95% confidence intervals (CIs) were calculated to aid interpretation.

## Results

Demographic and obstetric characteristics

A total of 50 patients with PPCM and LVEF below 45% were included. Group A (n=25) received bromocriptine with standard therapy, and Group B (n=25) received standard therapy alone. Age distribution was similar between groups (p = 0.976), and parity did not differ significantly (p = 0.13). Gestational age at presentation was significantly lower in Group A than in Group B (33.76 ± 7.84 vs 37.72 ± 1.57 weeks; p = 0.017), with a mean difference of -3.96 weeks (95% CI, -7.25 to -0.67). Four patients in Group A presented before 25 weeks of gestation, whereas none in Group B did. BMI was also lower in Group A (22.49 ± 3.10 vs 24.01 ± 1.75 kg/m^2^; p = 0.037), with a mean difference of -1.526 kg/m^2^ (95% CI, -2.97 to -0.08). These baseline differences were clinically important, were considered potential confounders, and are summarized in Table 1.

**Table 1:** Participant demographics and obstetric characteristics Values are expressed as number (%) unless marked as mean ± standard deviation (SD). Categorical variables were analyzed using the chi-square test or Fisher's exact test, and continuous variables were analyzed using the independent samples Student's t-test. A p-value less than 0.05 was considered statistically significant. *Statistically significant.

Parameter	Category	Group A: bromocriptine + standard therapy (n=25)	Group B: standard therapy (n=25)	P value
Age (years)	<25	10 (40%)	9 (36%)	0.976
	25-30	10 (40%)	10 (40%)	
	>30	5 (20%)	6 (24%)	
	Mean ± SD	26.36 ± 4.38	26.40 ± 4.82	
Parity	Primigravida	5 (20%)	11 (44%)	0.13
	Multigravida	20 (80%)	14 (56%)	
Gestational age at presentation (weeks)	<25	4 (16%)	0 (0%)	0.017*
	25-35	6 (24%)	2 (8%)	
	>35	15 (60%)	23 (92%)	
	Mean ± SD	33.76 ± 7.84	37.72 ± 1.57	
Body mass index (kg/m^2^)	<20	4 (16%)	0 (0%)	0.037*
	20.01-25.00	15 (60%)	17 (68%)	
	>25	6 (24%)	8 (32%)	
	Mean ± SD	22.49 ± 3.10	24.01 ± 1.75	

Clinical profile and medical history

Comorbidity distribution differed between the two groups (p = 0.033). Group A included patients with acute parenchymal renal disease, anemia, chronic hypertension, chronic nephritis, gestational diabetes on insulin, non-specific presentation, non-specific presentation with twins, hypothyroidism, and twin pregnancy. Group B included a higher recorded prevalence of hypothyroidism. Presenting complaints, past history, and family history were not statistically different between groups. The clinical profile and medical history variables are summarized in Table 2.

**Table 2:** Clinical profile and medical history of study participants Comparisons were performed using the chi-square test or Fisher's exact test, as appropriate. A p-value less than 0.05 was considered statistically significant. NSP: non-specific presentation; PPCM: peripartum cardiomyopathy.

Parameter	Category	Group A: bromocriptine + standard therapy (n=25), n (%)	Group B: standard therapy (n=25), n (%)	P value
Comorbidity	Acute parenchymal renal disease	1 (4%)	0 (0%)	0.033*
	Anemia	2 (8%)	0 (0%)	
	Chronic hypertension	1 (4%)	0 (0%)	
	Chronic nephritis	1 (4%)	0 (0%)	
	Gestational diabetes on insulin	3 (12%)	0 (0%)	
	Hypothyroidism	0 (0%)	8 (32%)	
	Non-specific presentation	4 (16%)	4 (16%)	
	NSP/twins with hypothyroidism	1 (4%)	0 (0%)	
	Twins	1 (4%)	0 (0%)	
	Nil	11 (44%)	13 (52%)	
Presenting complaints	Breathlessness	8 (32%)	2 (8%)	0.063
	Breathlessness with anemia	1 (4%)	0 (0%)	
	Palpitations	1 (4%)	0 (0%)	
	Nil	15 (60%)	23 (92%)	
Past history	Hypothyroidism	3 (12%)	0 (0%)	0.114
	Peripartum cardiomyopathy	1 (4%)	0 (0%)	
	Nil	21 (84%)	25 (100%)	
Family history	Myocardial infarction in father	0 (0%)	2 (8%)	0.078
	Myocardial infarction in mother	0 (0%)	2 (8%)	
	Stroke in father	0 (0%)	2 (8%)	
	Nil	25 (100%)	19 (76%)	

Baseline laboratory and biochemical parameters

Peripheral smear findings were comparable between groups (p = 0.19). Urine albumin distribution differed significantly (p = 0.006), with higher grades of albuminuria more frequent in Group B. Urine sugar, sodium, potassium, fasting blood sugar, HbA1c, troponin, and creatine phosphokinase-myocardial band (CPK-MB) did not differ significantly. Lipid profile abnormalities were more frequent in Group A (p = 0.002), whereas elevated liver transaminases were recorded only in Group B (p = 0.003). Mean thyroid-stimulating hormone (TSH) was lower in Group A than in Group B (2.34 ± 0.71 vs 3.71 ± 1.07 uU/mL; p < 0.001), and the difference in thyroid-related variables was considered another potential confounder. Baseline laboratory and biochemical parameters are summarized in Table 3.

**Table 3:** Baseline laboratory and biochemical parameters of study participants Values are expressed as number (%) unless marked as mean ± standard deviation (SD). Categorical variables were analyzed using the chi-square test or Fisher's exact test, and continuous variables were analyzed using the independent samples Student's t-test. A p-value less than 0.05 was considered statistically significant. The symbols +, ++, and +++ denote increasing concentration levels. SGOT: serum glutamic-oxaloacetic transaminase; SGPT: serum glutamic-pyruvic transaminase; TSH: thyroid-stimulating hormone; CPK-MB: creatine phosphokinase-myocardial band

Parameter	Category	Group A: bromocriptine + standard therapy (n=25)	Group B: standard therapy (n=25)	P value
Peripheral smear (CHG/PS)	Dimorphic anemia	1 (4%)	5 (20%)	0.190
	Hypochromic microcytic anemia	1 (4%)	0 (0%)	
	Microcytic anemia	1 (4%)	0 (0%)	
	Normal	22 (88%)	20 (80%)	
Urine albumin	+	4 (16%)	2 (8%)	0.006*
	++	0 (0%)	10 (40%)	
	+++	1 (4%)	0 (0%)	
	Loaded	2 (8%)	0 (0%)	
	Nil	18 (72%)	13 (52%)	
Urine sugar	+	0 (0%)	0 (0%)	1.000
	++	1 (4%)	0 (0%)	
	+++	0 (0%)	0 (0%)	
	Loaded	0 (0%)	0 (0%)	
	Nil	24 (96%)	25 (100%)	
Serum sodium (mEq/L)	Mean ± SD	137.8 ± 3.488	139.2 ± 4.359	0.216
Serum potassium (mEq/L)	Mean ± SD	3.812 ± 0.639	3.768 ± 0.256	0.751
Fasting blood sugar (mg/dL)	Mean ± SD	92.12 ± 13.286	91.56 ± 11.644	0.875
HbA1c (%)	<5	12 (48%)	8 (32%)	0.304
	>5	13 (52%)	17 (68%)	
	Mean ± SD	5.368 ± 0.726	5.596 ± 0.822	
Lipid profile	Hypercholesterolemia	3 (12%)	0 (0%)	0.002*
	Hypertriglyceridemia	7 (28%)	0 (0%)	
	Normal	15 (60%)	25 (100%)	
Liver function test	Elevated SGOT/SGPT	0 (0%)	9 (36%)	0.003*
	Normal	25 (100%)	16 (64%)	
TSH (uU/mL)	Mean ± SD	2.342 ± 0.707	3.712 ± 1.067	<0.001*
Troponin (ng/mL)	Mean ± SD	0.0264 ± 0.0196	0.0268 ± 0.0221	0.946
CPK-MB (IU/mL)	Mean ± SD	16.24 ± 7.401	15.8 ± 2.291	0.778

Cardiac assessment

Electrocardiographic findings differed between groups (p = 0.033), with sinus tachycardia recorded in Group A only, whereas ventricular premature complexes were present in both groups. Baseline LVEF distribution and mean LVEF did not differ significantly (38.36 ± 2.40% in Group A vs 39.00 ± 1.19% in Group B; p = 0.238). Chest radiographic findings differed significantly (p = 0.004), with cardiomegaly and increased vascular markings recorded only in Group A.

At discharge, mean LVEF was 47.52 ± 2.52% in Group A and 46.56 ± 2.82% in Group B (p = 0.210), with a mean between-group difference of 0.96 percentage points (95% CI, -0.56 to 2.48). The discharge comparison, therefore, did not rule out a clinically relevant difference. At one month, mean LVEF was 57.00 ± 1.71% in Group A and 49.84 ± 1.38% in Group B (p < 0.001), with a mean between-group difference of 7.16 percentage points (95% CI, 6.28 to 8.04). Cardiac assessment findings, including serial LVEF results, are summarized in Table 4.

**Table 4:** Cardiac assessment of study participants Values are expressed as number (%) unless marked as mean ± standard deviation (SD). Categorical variables were analyzed using the chi-square test or Fisher's exact test, and continuous variables were analyzed using the independent samples Student's t-test. A p-value less than 0.05 was considered statistically significant. LVEF: left ventricular ejection fraction.

Parameter	Category	Group A: bromocriptine + standard therapy (n=25)	Group B: standard therapy (n=25)	P value
Electrocardiogram	Sinus tachycardia	6 (24%)	0 (0%)	0.033*
	Ventricular premature complexes	5 (20%)	6 (24%)	
	Within normal limits	14 (56%)	19 (76%)	
Echocardiography (baseline LVEF%)	<35%	3 (12%)	0 (0%)	0.238
	>35%	22 (88%)	25 (100%)	
	Mean ± SD	38.36 ± 2.396	39.00 ± 1.190	
Chest X-ray	Cardiomegaly	5 (20%)	0 (0%)	0.004*
	Increased vascular markings	4 (16%)	0 (0%)	
	Normal	16 (64%)	25 (100%)	
LVEF at discharge (%)	<45%	4 (16%)	11 (44%)	0.21
	>45%	21 (84%)	14 (56%)	
	Mean ± SD	47.52 ± 2.519	46.56 ± 2.815	
LVEF after one month (%)	<45%	0 (0%)	0 (0%)	<0.001*
	>45%	25 (100%)	25 (100%)	
	Mean ± SD	57.00 ± 1.708	49.84 ± 1.375	

Obstetric and neonatal outcomes

Gestational age at delivery differed significantly between groups (p = 0.031), with preterm delivery more frequent in Group A. Mode of delivery was not significantly different (p = 0.324). Birth weight was lower in Group A (2.43 ± 1.13 vs 3.07 ± 0.41 kg; p = 0.01), with a mean difference of -0.64 kg (95% CI, -1.13 to -0.15). Apgar score distributions at one minute and five minutes differed between the groups (p < 0.001 for both). Nevertheless, because the Group B distributions were uniform and the groups differed in gestational age at delivery and birth weight, these findings were interpreted cautiously and were not attributed to bromocriptine. Neonatal intensive care unit admission was similar between groups (44% in Group A vs 32% in Group B; p = 0.56). Obstetric and neonatal outcomes are summarized in Table 5.

**Table 5:** Obstetric and neonatal outcomes Values are expressed as number (%) unless marked as mean ± standard deviation (SD). Group differences were evaluated using the chi-square test or Fisher's exact test for categorical variables and the independent samples Student's t-test for continuous variables. A p-value less than 0.05 was considered statistically significant. The Apgar values were verified against the original obstetric, delivery, and neonatal source records. Nevertheless, the uniform Group B distribution should be interpreted cautiously and should not be used to infer a treatment-related neonatal effect. NICU: neonatal intensive care unit.

Parameter	Category	Group A: bromocriptine + standard therapy (n=25)	Group B: standard therapy (n=25)	P value
Gestational age at delivery (weeks)	<25	4 (16%)	0 (0%)	0.031*
	25-35	3 (12%)	2 (8%)	
	>35	18 (72%)	23 (92%)	
	Mean ± SD	34.44 ± 7.52	37.96 ± 1.594	
Mode of delivery	Induced abortion	1 (4%)	0 (0%)	0.324
	Normal vaginal delivery	14 (56%)	14 (56%)	
	Lower segment cesarean section	8 (32%)	11 (44%)	
	Spontaneous expulsion	2 (8%)	0 (0%)	
Birth weight (kg)	<2.50	9 (36%)	2 (8%)	0.01*
	2.50-3.00	9 (36%)	11 (44%)	
	>3.00	7 (28%)	12 (48%)	
	Mean ± SD	2.43 ± 1.126	3.072 ± 0.411	
Apgar score (one minute)	0	4 (16%)	0 (0%)	<0.001*
	5	2 (8%)	25 (100%)	
	6	3 (12%)	0 (0%)	
	7	4 (16%)	0 (0%)	
	8	12 (48%)	0 (0%)	
Apgar score (five minutes)	0	4 (16%)	0 (0%)	<0.001*
	7	3 (12%)	0 (0%)	
	8	2 (8%)	0 (0%)	
	9	13 (52%)	0 (0%)	
	10	3 (12%)	25 (100%)	
NICU admission	Yes	11 (44%)	8 (32%)	0.56
	No	14 (56%)	17 (68%)	

## Discussion

This study evaluated early LVEF recovery among patients with PPCM treated with standard therapy with or without low-dose one-week bromocriptine. The principal finding was that both groups improved, and Group A had higher mean LVEF at one month. However, the finding must be interpreted as an association rather than proof of treatment effect because the study was observational, nonrandomized, small, and affected by important baseline imbalances.

The standard therapy issue is central to interpretation. In PPCM, background therapy must be adapted to pregnancy status, postpartum status, lactation, blood pressure, renal function, and thromboembolic risk. General HFrEF guidance supports multidrug therapy when safe and appropriate, but pregnancy and lactation can restrict or delay use of several drug classes [2,3]. Therefore, the term standard therapy in this study should be understood as pregnancy/postpartum-compatible therapy available during the study period, not as confirmation that all patients received contemporary full four-pillar HFrEF therapy. Because patient-level drug names, doses, duration, and adherence were not available, residual treatment differences between groups cannot be excluded.

The bromocriptine regimen also requires cautious interpretation. Group A received 2.5 mg once daily for one week. Published PPCM studies and protocols include both short-course and prolonged regimens, and the randomized German protocol specified anticoagulant therapy during the period of bromocriptine exposure [12,13]. The present study therefore evaluates a low cumulative dose strategy and cannot be generalized to prolonged bromocriptine protocols or to settings without careful thromboembolic risk assessment. The lack of patient-level anticoagulation data is a major limitation when assessing safety.

The direction and magnitude of the one-month LVEF difference may also be influenced by baseline differences. Group A had a wider gestational-age distribution, included patients presenting before 25 weeks of gestation, had a lower mean BMI, and had more abnormal electrocardiographic and chest radiographic findings at baseline. Group B had more recorded cases of hypothyroidism, higher TSH values, and more liver function test abnormalities. These imbalances may reflect different disease phenotypes, hemodynamic load, timing of diagnosis, nutritional status, or comorbidity burden. Without multivariable adjustment or stratified analyses, it is not possible to determine how much of the observed one-month LVEF difference was related to bromocriptine rather than baseline patient characteristics.

The discharge LVEF comparison illustrates the limitations of relying only on p-values in this small cohort. Although the categorical difference in LVEF below 45% at discharge appeared clinically relevant, the p-value was not statistically significant, and the confidence interval for the mean discharge LVEF difference crossed zero. This study was not adequately powered to exclude a clinically meaningful difference at discharge, and the one-month result should be viewed in the context of multiple unadjusted comparisons.

Neonatal outcomes require particularly cautious interpretation. Group A delivered at a lower gestational age and had lower birth weight, which are strong determinants of neonatal condition independent of bromocriptine. However, the uniform values in Group B, together with the between-group differences in gestational age at delivery and birth weight, require cautious interpretation. Therefore, the observed Apgar differences were not used to infer treatment-related neonatal benefit or harm.

Breastfeeding is another clinically important outcome. Bromocriptine suppresses lactation, and this has implications for maternal counseling, infant feeding, patient preference, and ethical consent. The present dataset did not capture breastfeeding intention, lactation counseling, alternative feeding plans, or infant feeding outcomes. This omission limits assessment of maternal-infant consequences of the intervention.

The present findings are consistent with the broader hypothesis that prolactin inhibition may be associated with improved ventricular recovery in PPCM, but they are less definitive than randomized or protocolized studies [10-15]. Echocardiographic assessment followed published chamber quantification recommendations [17], and earlier consensus documents and reviews support the diagnostic framework used for PPCM [18,19]. Observational studies from the United States and global systematic reviews have emphasized broad heterogeneity in clinical presentation and recovery [20-22], and population-based data support an association between hypertensive disorders and PPCM [23]. Additional pooled analyses of bromocriptine have suggested possible short-term improvement in LVEF, but the certainty of evidence remains limited [24]. These external data reinforce the need to account for phenotype and baseline risk before attributing recovery differences to a single drug.

Safety considerations remain important. Bromocriptine has been used for lactation suppression and prolactin-mediated conditions, but thrombotic and cardiovascular complications have been reported, particularly in the postpartum setting [25,26]. Historical descriptions and long-term outcome analyses show that PPCM recovery and prognosis often evolve over months to years rather than over one month [27,28]. Biomarker studies suggest that troponin and other markers may help identify persistent dysfunction in selected patients, but the present study did not demonstrate significant baseline troponin differences between groups [29].

This study has several strengths, including prospective enrollment from a tertiary care setting, direct comparison of two treatment strategies, and serial LVEF assessment. The limitations are substantial: nonrandomized treatment allocation, no documented allocation concealment or blinding, no a priori sample size calculation, small sample size, single-center design, one-month follow-up, lack of multivariable adjustment, multiple unadjusted comparisons, incomplete patient-level documentation of heart failure medications and anticoagulation, missing prolactin data. Additional limitations included incomplete lactation and breastfeeding data, broader-than-classical gestational inclusion, baseline imbalances in gestational age and body mass index, and the uniform Apgar score distribution in Group B, which limits interpretation despite source-record verification. These limitations substantially restrict causal inference and should be addressed in future studies.

## Conclusions

In this prospective comparative observational study, both treatment groups demonstrated early improvement in left ventricular function during follow-up. Patients who received adjunctive low-dose bromocriptine experienced a greater improvement in LVEF at one month. However, because of the nonrandomized study design, baseline differences between the groups, incomplete documentation of background medical therapy, and the relatively short follow-up period, this finding cannot be attributed solely to bromocriptine. Neonatal outcomes should also be interpreted with caution, as they may have been influenced by prematurity and differences in maternal disease severity at baseline. Future well-designed prospective studies with larger sample sizes, standardized contemporary heart failure management, comprehensive documentation of anticoagulation and lactation counseling, source-verified neonatal data, appropriate statistical adjustment for confounding factors, and longer follow-up are needed to better define the role of bromocriptine in the management of peripartum cardiomyopathy.
